# Context‐Dependent Effects of Ivermectin Residues on Dung Insects: Interactions With Environmental Stressors, Size, and Sex in a Sepsid Fly (*Sepsis neocynipsea*)

**DOI:** 10.1002/ece3.71929

**Published:** 2025-08-08

**Authors:** Jill Walker, Benjamin J. Mathews, Patrick T. Rohner

**Affiliations:** ^1^ Department of Ecology, Behavior, and Evolution University of California San Diego La Jolla California USA

**Keywords:** desiccation stress, Diptera, ecotoxicology, ivermectin, Sepsidae, sex, temperature stress

## Abstract

Coprophagous insects frequently encounter veterinary medication residues that are excreted unmetabolized in livestock dung. These residues often negatively affect insect survival, reproduction, and ecosystem services and may contribute to the rapid decline in insect populations. Ivermectin is an antiparasitic drug widely used to treat parasites in livestock. While it has long been recognized that ivermectin residues affect insect survival, the potential interactive effects between ivermectin exposure and other ecologically relevant abiotic stressors remain poorly understood. Here, we study these effects in the black scavenger fly *Sepsis neocynipsea* , which depends on cow dung for reproduction. Using a fully factorial experimental design, we test whether the effects of ivermectin exposure on adult survival interact with heat and desiccation stress and whether the severity of these effects depends on an individual's size and sex. We found that ivermectin exposure had strong negative impacts on adult survival overall, but that mortality was approximately three times higher in females compared to males. The combination of ivermectin exposure, heat, and desiccation stress drastically reduced survival. Interestingly, individuals simultaneously exposed to heat and ivermectin stress survived better—on average— than individuals only exposed to ivermectin, suggesting potential hormetic effects. Taken together, our findings highlight how the complex interactions between veterinary pharmaceuticals and abiotic stressors could drive changes in coprophagous insect populations and their ecological functions.

## Graphical abstract

Veterinary medication residues in livestock dung, such as the antiparasitic drug ivermectin, can negatively affect insects that rely on dung for reproduction, impacting survival, reproduction, and ecosystem functions. This study on the dung fly *Sepsis neocynipsea* reveals that ivermectin exposure significantly reduces adult survival, particularly in females and larger individuals, and interacts with heat stress to produce non‐additive effects, suggesting cross‐resistance. These findings underscore the complex interplay between insecticides and abiotic stressors in driving changes in insect populations and ecosystem dynamics.

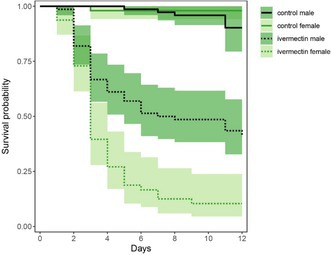

## 1 Introduction

The environmental impacts of agricultural chemicals, including pesticides and veterinary pharmaceuticals, are a major concern in conservation and environmental management (Zhou et al. 2025). Antiparasitic drugs are a large component of the animal health market, accounting for €7 billion in annual sales as of 2018 (Selzer and Epe 2021). These drugs treat an increasingly broad spectrum of endo‐ and ectoparasites, greatly benefiting animal and human health (Crump 2017). Despite these benefits, there have been concerns over the ecological impact of antiparasitic drugs when they enter ecosystems. Even low doses of broad‐spectrum antiparasitics can negatively affect off‐target organisms in the environment (Gandara et al. 2024). This can be especially problematic for coprophagous (i.e., dung‐eating) insects that are often in contact with unmetabolized chemical residues found in the feces of treated livestock.

The impact of antiparasitic residues has been heavily studied in coprophagous insects. This ecological guild incorporates members from several families of dung beetles and dung flies and plays important roles in regulating the decomposition of fecal matter, especially in agricultural contexts (Kavanaugh and Manning 2020; Losey and Vaughan 2006; Skidmore 1991). Through consuming, burying, aerating, and fragmenting dung, coprophagous insects directly and indirectly facilitate fecal decomposition, often driving the local microbial, fungal, and invertebrate diversity (Stevenson and Dindal 1987). However, the reliance of coprophagous insects on dung as a food source frequently exposes them to chemicals excreted in vertebrate dung. One such chemical is ivermectin—a broad‐spectrum antiparasitic drug often used to treat nematode, mite, and lice infections in livestock, pets, and humans (Crump 2017). As much as 80%–90% of an ivermectin dose can be excreted through feces, and, due to its chemical stability, residues can remain in the environment for weeks (Alvinerie et al. 1999; Herd et al. 1996; Madsen et al. 1990). Because ivermectin is a broad‐spectrum antiparasitic that acts on a wide range of arthropods and nematodes (El‐Saber Batiha et al. 2020; McKellar 1997; Puniamoorthy et al. 2014), environmental residues can negatively impact the coprophagous invertebrates that rely on livestock feces for food and reproduction, thereby greatly impairing ecosystem functioning (Correa et al. 2022; Jochmann and Blanckenhorn 2016; Kavanaugh and Manning 2020; Madsen et al. 1990; Verdú et al. 2018).

Although ivermectin can be directly lethal to coprophagous insects by disrupting molting, growth, and reproduction (McKellar 1997; Pérez‐Cogollo et al. 2015; Puniamoorthy et al. 2014; Rodríguez‐Vivas et al. 2020; van Koppenhagen et al. 2020), its effects are not universally fatal (Conforti et al. 2018; Jochmann and Blanckenhorn 2016; Schmidt 1983). The environmental and physiological factors that mediate susceptibility to ivermectin, however, remain poorly understood. Recent studies suggest that environmental conditions, such as heat stress, may interact with ivermectin exposure to amplify its effects (Bueno et al. 2023; González‐Tokman et al. 2022; Sirois‐Delisle and Kerr 2022). Likewise, intrinsic traits like body size or sex—which are known to influence sensitivity to other chemicals in other taxa (e.g., Rathman et al. 1992; Zhang et al. 2019)—may also shape ivermectin responses in coprophagous insects. Yet, these interactions have not been systematically explored. Here, we begin to investigate how such factors modulate ivermectin sensitivity in black scavenger flies.

Black scavenger flies (Sepsidae) are a functionally important group of coprophagous insects abundant in temperate and alpine grasslands. Despite their small size, ranging from 2 to 6 mm in body length, they play important roles as detritivores, pollinators, and a food source for other invertebrates (Pont and Meier 2002; Rohner et al. 2014, 2019). Most sepsid species rely on vertebrate excrement for reproduction, with adult females of many species laying their eggs on the surface of fresh cow dung. The larvae hatch and feed on the dung until they pupate either inside or near the cow pat. Cow dung is essential not only as a site for oviposition but also as a crucial food source for egg production in adult females (Pont and Meier 2002). Given their ecological role and dependence on vertebrate dung across their entire lifecycle, sepsids are an ideal system for studying the ecosystem effects of veterinary antiparasitic pharmaceuticals and their context dependency.

Previous studies have shown that sepsid larvae are very sensitive to ivermectin. Larval sepsids exposed to ivermectin residues in cow dung show high rates of mortality, even at very low and ecologically relevant concentrations (Blanckenhorn et al. 2013; Puniamoorthy et al. 2014). Adult flies, which frequently visit dung pats for oviposition, mating, and feeding, are also significantly affected by ivermectin exposure. Adults feeding on cow dung containing the antiparasitic show reduced survival, fecundity, and fertility (Conforti et al. 2018). While interspecies differences in ivermectin tolerance have been documented, little is known about how ivermectin exposure affects individuals within a species under varying environmental conditions. This is of particular concern because temperate agricultural landscapes are predicted to experience increasing temperature and desiccation stress (Yang et al. 2024), which could potentially add to the negative effects of pharmacological residues. For instance, a recent study in the yellow dung fly suggests that exposure to high temperatures might exacerbate the negative effects of ivermectin residues (González‐Tokman et al. 2022). However, whether such interactive effects are widespread is still unclear (Halsch et al. 2023).

In addition to external environmental stressors, the effects of ivermectin exposure might also depend on an individual's endogenous features, such as its size or sex. A large body of research demonstrates that female insects are often more sensitive to nutritional conditions, possibly due to the costly development of eggs and ovaries (Rohner et al. 2018; Stillwell et al. 2010; Teder and Tammaru 2005). Similar effects could be expected for the exposure to chemical residues in the environment. Given females' role in population growth rates, sex‐specific effects could further exacerbate (or reduce) the ecological effects of ivermectin exposure.

Here, we investigate how ivermectin exposure interacts with other ecological and endogenous factors in *Sepsis neocynipsea* Melander and Spuler, 1917 (Diptera: Sepsidae)—a species with a broad Holarctic distribution common in North American grasslands (Pont and Meier 2002). Using a fully factorial design, we test for interactive effects between ivermectin exposure, heat, and desiccation stress, as well as size‐ and sex‐specific effects. We hypothesized that ecological and pharmaceutical stressors would act synergistically on mortality—that is, that their combined effects would exceed the sum of their individual impacts. As expected, ivermectin exposure strongly reduced adult survival. The combination of ivermectin and desiccation stress increased mortality, although the effects were largely additive rather than synergistic. Unexpectedly, the combination of ivermectin and heat stress led to lower mortality than that predicted from their independent effects, suggesting an antagonistic interaction. These results highlight that the effects of veterinary pharmaceutical residues on dung insect communities are likely to be highly context‐dependent in natural environments.

## 2 Methods

### 2.1 Experimental Design

We collected wild *Sepsis neocynipsea* females on a cow pasture in Bloomington, Indiana, USA and brought them into the lab at the University of California San Diego. Each female was placed into a 50 mL conical (Falcon) tube that contained 25 g of previously frozen cow dung. Tubes were incubated at room temperature for 3 weeks, allowing females to lay eggs and their offspring to emerge as adults. Once offspring emerged, we verified taxon identification following Pont and Meier (2002). The offspring of 30 female *Sepsis neocynipsea* were then combined in a 1.9 L plastic container to establish a large outbred laboratory colony. This colony was provided with previously frozen cow dung, water, and sugar at 21°C (following standard laboratory procedures; see e.g., Rohner et al. 2016).

**FIGURE 1:**
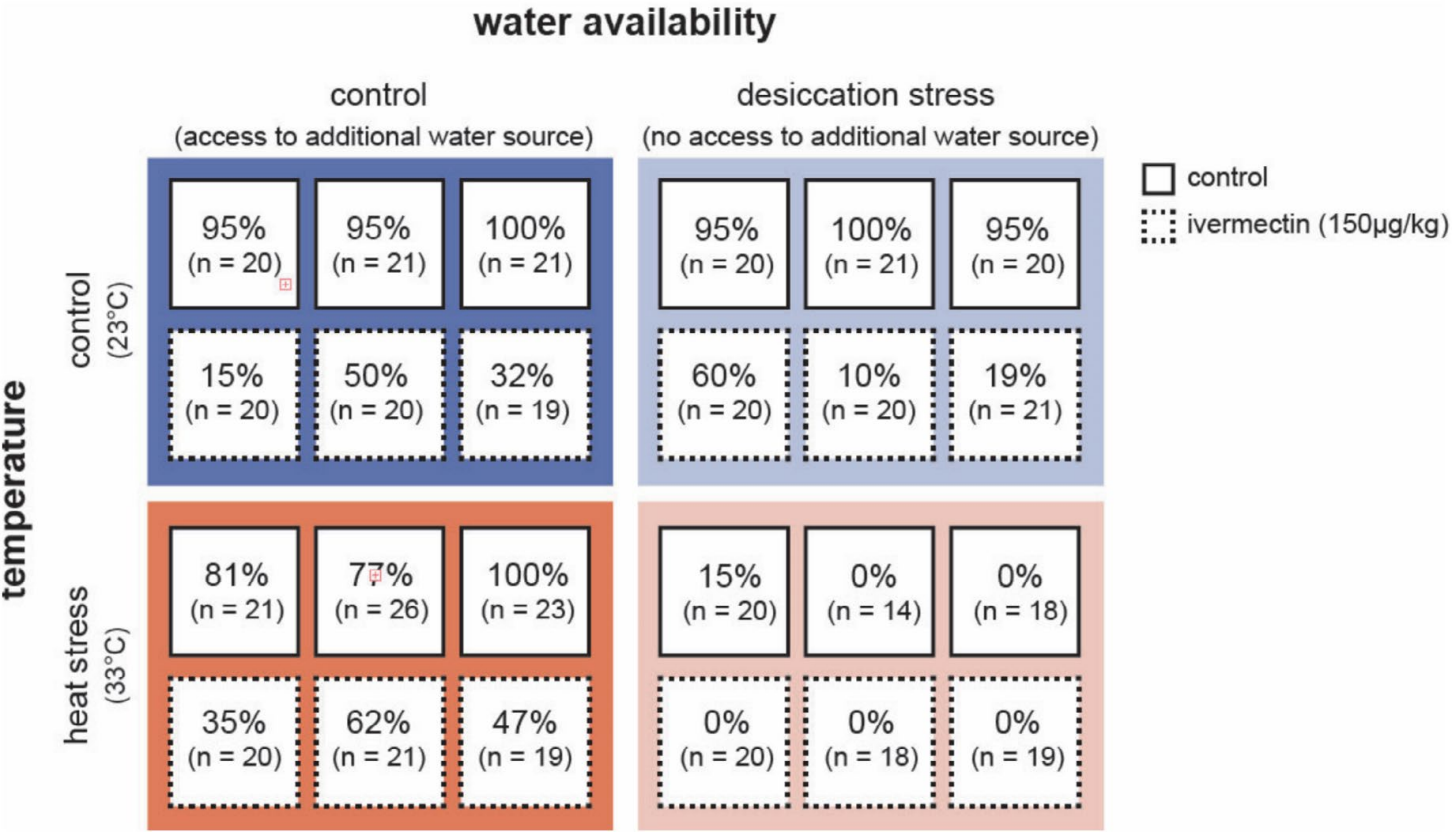
Graphical representation of the experimental design. The experiment used a fully‐factorial design that crossed two temperature treatments (23°C vs. 33°C) with two desiccation treatments (with vs. or without an additional water source), as well as an ivermectin treatment (acetone control vs. ivermectin). For each of the treatment combinations, there were three replicates (indicated by squares). The total number of individuals (*n*) as well as the observed survivorship after the experiment was terminated (in percent) are given per replicate.

To assess the context‐dependence of ivermectin exposure on adult survival, we conducted replicated laboratory trials with a fully factorial design that incorporated heat stress, desiccation stress, and ivermectin treatments (experimental design shown in Figure 1). Each replicate trial consisted of a 1.9 L plastic container equipped with a small 30 mL ramekin (deli cup) containing ca. 1.5 g of sugar. Between 20 and 26 adult flies that were at least 2 weeks old were randomly assigned to each container and transferred using an aspirator. In total, we used 24 replicate containers for the duration of this experiment.

To implement the temperature treatment, half of the replicate containers were placed in an incubator (Caron Insect Growth Chamber, 7340‐25‐1) set to a constant 23°C, and the other half in an incubator set to 33°C. To manipulate desiccation stress, we manipulated the presence of an additional water source. Half of the containers contained a 30 mL lidded plastic cup filled with water, with a cotton string threaded through the lid to wick water to the surface for fly access. The remaining containers lacked an external water source, and dung (see below) represented the only source of moisture.

To assess the effects of ivermectin, we provided each container with a 59 mL cup filled with 20 g of previously frozen cow dung that either did or did not contain ivermectin. In the control treatment, 500 μL of pure acetone was mixed into the dung, whereas in the ivermectin treatment, dung was mixed with 500 μL of acetone containing 3 μg of ivermectin (as each cup contained 20 g of dung, this resulted in an ivermectin concentration of 150 μg/kg wet weight). This concentration is comparable to field estimates of fecal ivermectin concentration at the excretion peak a few days after treatment. For instance, Lumaret et al. (2007) and Fernandez et al. (2009) measured ivermectin concentrations of 145 μg/kg and approximately 250 μg/kg in cow dung (estimates based on wet weight; see Liebig et al. 2010). For both treatments, we allowed acetone (which is used as solvent) to evaporate from the dung for 5 h before collecting flies to place into their respective containers.

Each of the eight possible combinations of the three binary treatments (2 ivermectin treatments × 2 temperature treatments × 2 desiccation treatments) was replicated three times, leading to a total of 24 replicates and a total of 482 flies used in the experiment (see Figure 1). The experiment was run in two different temporal blocks. The first block contained one replicate per treatment combination, while the second block contained two replicate containers.

### 2.2 Adult Survival, Sex, and Size Estimation

We collected dead flies every 24 h to record mortality and sexed each individual based on the presence (or absence) of a hypopygium (genital clasper that is only present in males) and conspicuous spines on the forefemur that are only found in males (Baur et al. 2019; Rohner and Blanckenhorn 2018). The experiment was terminated when there were signs of late‐stage pupae in the control treatments. To generate an estimate of overall body size, we removed the left and right wings of all individuals, embedded them in glycerol on a glass slide, and photographed them using a Pixelink camera (M20C‐CYL) mounted on a Leica M205 stereoscope. We then used ImageJ to measure the length of the second longitudinal wing vein as an estimate of adult body size (see Figure S1). The length of this wing vein is strongly correlated with other linear morphological traits and is thus a suitable body size estimate (see Table S1). Due to wing wear, size could not be estimated for all individuals. When measurements for the left and right sides were available, we used the mean for further analysis.

### 2.3 Statistical Analyses

We analyzed adult survival using a Cox proportional hazards mixed‐effects model fitted via maximum likelihood, as implemented in the R package coxme (Therneau 2022). Fixed effects included wing length (as a proxy for body size), temperature, desiccation stress, ivermectin exposure, and sex, as well as all possible interactions among these variables. Wing length was mean‐centered prior to analysis. Experimental replicate (containers within temporal block) was included as a random intercept to account for non‐independence among individuals reared under the same conditions. Individuals that survived until the end of the experiment were treated as right‐censored observations.

The model including all main effects can be expressed as:

$$h_{i}\left(t\right)=h_{0}\left(t\right)\cdot \exp\left(\beta_{1}{\mathrm{WL}}_{i}+\beta_{2}T_{i}+\beta_{3}D_{i}+\beta_{4}I_{i}+\beta_{5}S_{i}+b_{j}\right)+\varepsilon_{i}$$

where $h_{i}\left(t\right)$ is the hazard (i.e., the instantaneous risk of death) of individual i at time t, $h_{0}\;\left(t\right)$ is the baseline hazard function, WL is mean‐centered wing length, T is the effect of temperature, D is the desiccation treatment, I is ivermectin exposure, and S represents sex. The random effect of the replicate container is represented by $b_{j}$ and the residual error term is indicated with ε. We initially fitted a full model including all two‐ to five‐way interactions, then sequentially removed nonsignificant interaction terms using backward elimination (Neter et al. 1985). Main effects for wing length, sex, temperature, desiccation, and ivermectin exposure were retained throughout the analysis as they represented hypotheses of a priori interest.

## 3 Results

**FIGURE 2:**
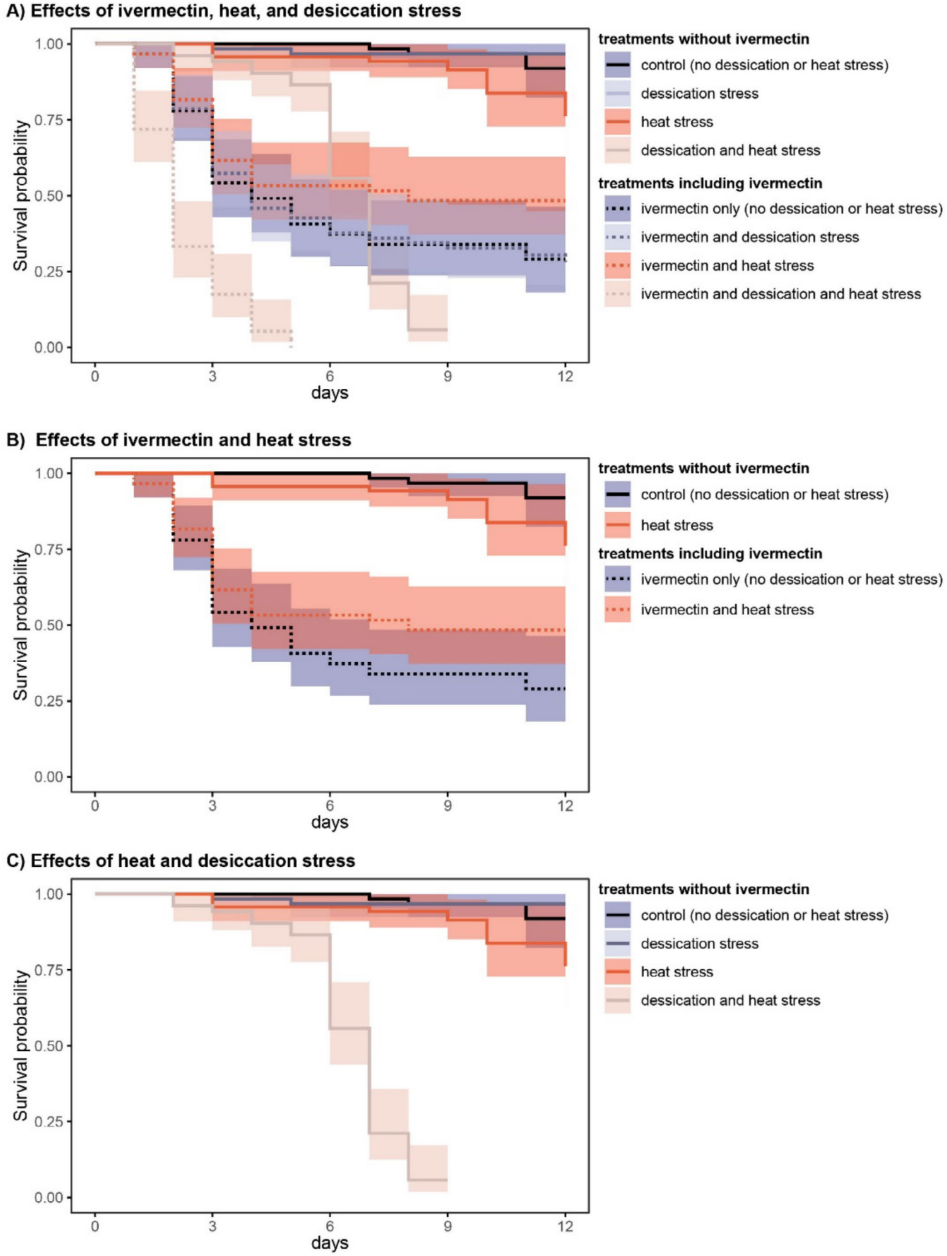
Effect of ivermectin in combination with other ecological stressors on adult survival of *Sepsis neocynipsea*. Plots show survival probability with time and associated 95% confidence limits. Panel (A) shows the combined effects of desiccation and heat stress in combination with ivermectin exposure. Treatment combinations including ivermectin exposure are indicated with a hatched line. Panel (B) highlights the subset of treatments also shown in A that are associated with the interaction of ivermectin and heat stress. Panel (C) similarly shows the subset of treatments associated with desiccation and heat exposure.

To assess the effect of ivermectin exposure on adult survival, we exposed a total of 482 individual sepsid flies to six different treatment combinations. Across all treatments, 48.8% (235/482) of all individuals died in the course of the experiment (Figure 2A).

**FIGURE 3:**
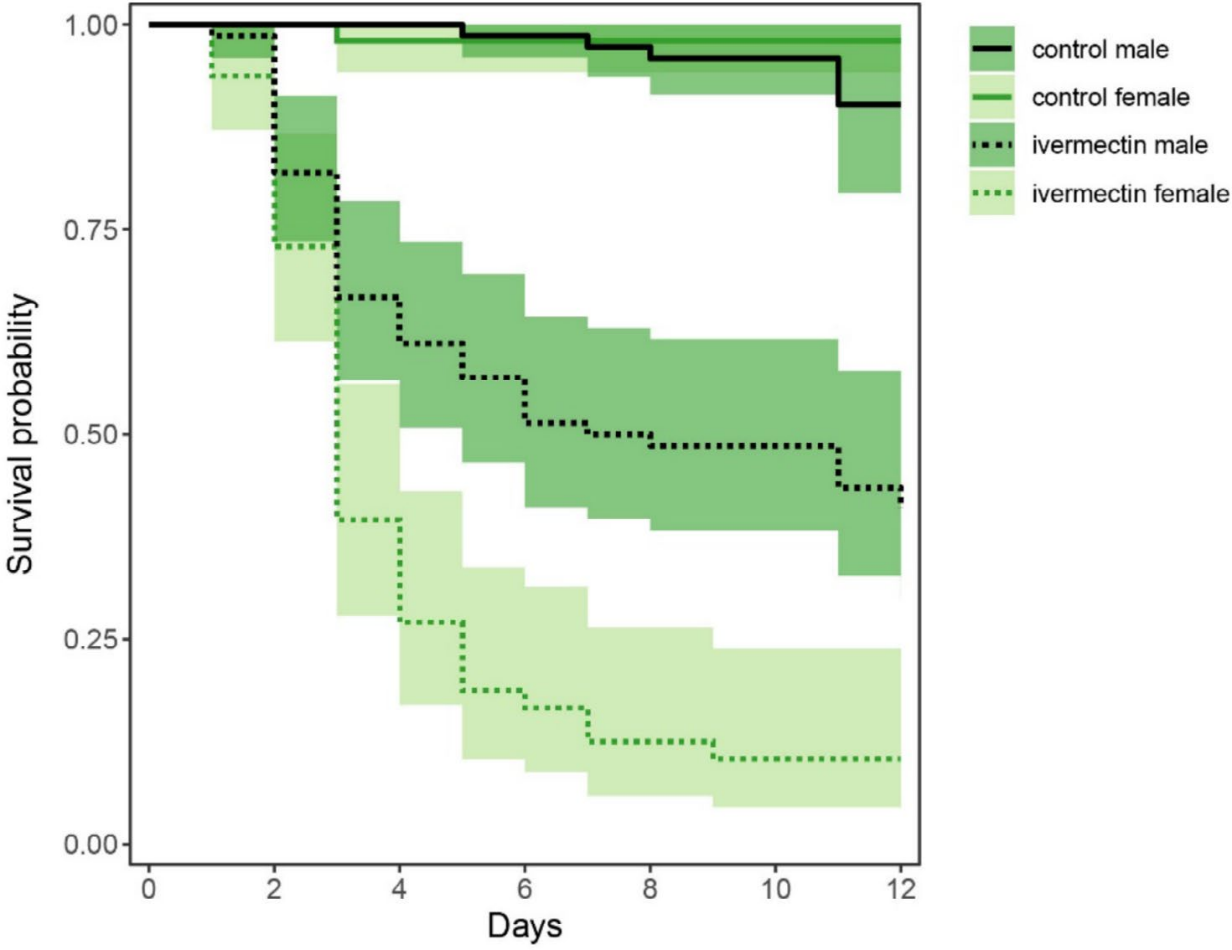
Sex‐specific effects of ivermectin exposure on survival. Plot shows survival probability with time and associated 95% confidence limits. To show the sex‐specific effects, we pooled the different treatments within each sex and ivermectin treatment combination. The analyses discussed in the main text (and the corresponding hazard rations) are based on the full model that took all treatment combinations into account.

Using a Cox mixed‐effects model, we found that ivermectin exposure had a very strong overall effect on survival across all treatments (ivermectin treatment main effect: HR = 74.85, *z* = 7.00, *p* < 0.001). Ivermectin was especially strong in females, who were about three times less likely to survive compared to males (sex‐by‐ivermectin treatment interaction: HR = 0.33, *z* = −3.00, *p* = 0.003, Table 1, Figure 3). While desiccation and heat stress had very strong synergistic effects on survival when combined simultaneously (desiccation‐by‐temperature stress interaction: HR = 11.87, *z* = 4.08, *p* < 0.001, Table 1), we did not find any significant synergistic interactions between either variable and ivermectin treatment. Instead, the combined effect of ivermectin and heat stress on survival was less than the sum of the individual impacts of ivermectin and heat stress (heat stress‐by‐ivermectin interaction: HR = 0.21, *z* = −2.27, *p* = 0.023, Table 1; Figure 2B). This pattern is evident in pairwise comparisons among treatments without additional desiccation stress. Individuals exposed to heat stress alone (without ivermectin) were 2.6 times more likely to die than control individuals kept at 23°C (although this difference was not statistically significant in this particular pairwise comparison: HR = 2.63, *z* = 1.32, *p* = 0.186). Ivermectin exposure at 23°C increased mortality 24‐fold compared to controls at the same temperature (HR = 23.8, *z* = 4.68, *p* < 0.001). However, individuals exposed to both ivermectin and high temperatures were only 16 times more likely to die compared to controls (HR = 15.9, *z* = 4.05, *p* < 0.001). Thus, heat stress appeared to reduce the lethality of ivermectin exposure relative to the control temperature.

**TABLE 1:** Cox mixed‐effects model fit by maximum likelihood (*n* = 466; 16 individuals with missing wing length excluded).

	Coefficient	SE	HR	*Z*	*p*
Wing length (mean‐centered)	1.67	1.00	5.33	1.67	0.095
Heat stress [23°C → 32°C]	1.21	0.67	3.35	1.80	0.072
Desiccation stress [control → desiccation]	0.01	0.46	1.01	0.03	0.980
Ivermectin treatment [control → antiparasitic exposure]	4.32	0.62	74.85	7.00	**< 0.001**
Sex [female → male]	0.12	0.32	1.13	0.38	0.700
Wing length × heat stress	−2.94	0.76	0.05	−3.87	**< 0.001**
Wing length × desiccation stress	1.61	0.72	5.03	2.23	**0.025**
Wing length × ivermectin treatment	−2.01	0.80	0.13	−2.53	**0.012**
Heat stress × ivermectin treatment	−1.58	0.70	0.21	−2.27	**0.023**
Ivermectin treatment × sex	−1.09	0.36	0.33	−3.00	**0.003**
Heat stress × desiccation stress	2.47	0.61	11.87	4.08	**< 0.001**

*Note:* Significant effects (*p* < 0.05) are indicated in bold.

Abbreviations: HR, hazard ratio; SE, standard error.

Effects of the various chemical and environmental stressors also depended on body size. Large individuals were more resistant to high temperatures and ivermectin exposure compared to smaller individuals (size‐by‐ivermectin interaction: HR = 0.13, *z* = −2.53, *p* = 0.012; size‐by‐temperature interaction: HR = 0.05, *z* = −3.87, *p* = 0.001, Table 1). However, larger individuals were more strongly affected by desiccation stress (size‐by‐desiccation interaction: HR = 5.03, *z* = 2.23, *p* = 0.025, Table 1). Larger individuals did therefore not have a survival advantage in all contexts.

## 4 Discussion

Dung insects are frequently exposed to unmetabolized veterinary pharmaceuticals (Gandara et al. 2024), but the degree to which this interacts with other ecological stressors remains unclear. We investigated how ivermectin exposure, heat, and desiccation stress impact the survival in adults of the sepsid fly *Sepsis neocynipsea* . Our results indicate that ivermectin exposure significantly increases mortality, though this effect varied by context. Mortality was notably higher among females and smaller individuals, while the lethal effects of ivermectin depended on temperature. Larger flies showed greater resilience to heat stress but were more vulnerable to desiccation. Overall, complex interactions among sex, body size, temperature, and ivermectin exposure can—depending on the specific conditions—modulate the mortality caused by ivermectin exposure. These findings have implications for the ecology of black scavenger flies in agricultural systems, particularly as these ecosystems are expected to face increasing pressures through pesticides, water availability, as well as temperature (Yang et al. 2024).

### 4.1 Interactions Between Different Ecological Stressors in Complex Environments

In natural environments, insects are exposed to varied, interacting stressors that are often not captured in experiments focused on exposure to singular stressors (Bueno et al. 2023; Rodrigues and Beldade 2020; Rohner and Moczek 2023). These interactions are difficult to predict but might contribute to the rapid decline of insect populations observed in the field. For example, in our experiment, heat and desiccation stress individually caused moderate reductions in survival, but their combination resulted in a severe decline (Figure 2C), illustrating the potential for strong synergistic effects. We hypothesized that similar interactions might occur between chemical residues and abiotic stressors. Previous research found that heat stress and ivermectin exposure act synergistically to reduce offspring survival in yellow dung flies (González‐Tokman et al. 2022), a distantly related group of flies that is also dependent on cow dung for reproduction. In contrast to this work, we did not find the expected synergistic interactions between ivermectin exposure and either of these major abiotic stressors in *S. neocynipsea* . Specifically, desiccation stress did not interact with ivermectin, and individuals simultaneously exposed to heat and ivermectin stress survived better than individuals only exposed to ivermectin. This unexpected finding may indicate a form of cross‐resistance (or hormesis), where exposure to one stressor confers increased resistance to another. Such pesticide‐induced hormetic effects have been found in other contexts and species (Guedes et al. 2022) and temperature has previously been shown to improve insecticide resistance in various insect pests (Bueno et al. 2023). For instance, in the brown planthopper, exposure to a sublethal dose of an insecticide leads to increased thermotolerance—a phenomenon likely mediated by cellular repair and maintenance mechanisms, potentially including the expression of heat shock proteins (Ge et al. 2013). Similar patterns have been found in dung beetles where ivermectin‐treated females increased the expression of the heat shock protein Hsp70 and males increased their antioxidant capacity (Villada‐Bedoya et al. 2021). If thermal responses indeed increase ivermectin resistance, the ancient adaptive response to heat could serve as an exaptation (sensu Gould and Vrba 1982) for handling exposure to ivermectin, which has only recently become widespread in agricultural environments. However, the extent to which such exaptation is likely requires further investigation. Overall, these findings suggest that examining the interactive effects of ivermectin and other environmental stressors is essential to better understand how antiparasitics impact dynamic and rapidly changing ecosystems.

### 4.2 Sex‐Specific Effects of Ivermectin Exposure on Adult Survival

Our findings indicate that females are about three times more likely to die from ivermectin exposure than males. These sex‐specific effects of ivermectin are potentially due to the distinct nutritional needs of females. In many insect species, including blowflies (Vogt et al. 1985), females feed on more protein‐rich food sources compared to males to support egg production. Because sepsid flies seem unable to differentiate between contaminated and ivermectin‐free cow dung (Blanckenhorn et al. 2013; Conforti et al. 2018), this could lead to an increased consumption of ivermectin dung in females, potentially explaining the sex difference in mortality when exposed to the same contaminated environment.

Alternatively, females may be more sensitive to chemical stresses due to their physiology. Previous studies indicate that females are often more vulnerable to nutrient stress than males (Teder and Tammaru 2005), and similar patterns could extend to chemical stress, although higher female mortality is not consistent across insect groups (e.g., Andreazza et al. 2020). Regardless of the underlying mechanism, increased female mortality is concerning, as the number of females in a population determines the overall potential population growth rate. Ivermectin‐driven reductions in female numbers could therefore significantly impact population dynamics and genetic diversity (Sutton et al. 2014). It is unclear, however, whether changes in the total number of females in a population (or a change in sex ratios) would lead to immediate consequences for ecosystem functioning. Ivermectin residues have previously been shown to affect rates of dung removal (Madsen et al. 1990), but the degree to which this effect is mediated by sepsids, as opposed to the much larger species of dung beetles or other large invertebrates, is poorly understood.

### 4.3 Interactions Between Ivermectin Exposure and Body Size

Large body size is often hypothesized to confer greater resistance to environmental stressors, including exposure to environmental toxins. For instance, larger insect species often exhibit greater tolerance to a given total amount of insecticides compared to smaller species (e.g., Faly et al. 2023; Nagloo et al. 2024). We found a similar pattern within species in that larger individuals exhibited lower mortality rates when exposed to ivermectin. As we did not experimentally control for individual ingestion rates, it is unclear whether this is mediated by physiology (i.e., an endogenously higher level of resistance) or whether size‐dependent foraging behaviors could mediate the effect. However, irrespective of the mechanism, size‐dependent mortality suggests that ivermectin exposure might impose increased positive directional selection on size in the field.

The body size‐dependence of ivermectin found within species contrasts with patterns previously documented across species. Puniamoorthy et al. (2014) measured the ivermectin dose at which 50% of exposed larvae die (i.e., LC_50_) and showed that resistance to ivermectin varies more than 500‐fold across 21 species of sepsids. They also showed that this surprising amount of variation is not correlated with the macroevolution of body size. This indicates that the evolution of body size and ivermectin resistance is largely decoupled across species. The relationship between size and resistance found in *S. neocynipsea* thus does not seem to extend to the macroevolutionary level. Whether similar size‐dependent effects hold within other species of sepsids remains to be tested.

In addition to higher ivermectin resistance, large individuals also had greater resistance to heat stress. Larger individual's resistance to high temperatures aligns with the findings of previous studies showing that larger individual insects have greater heat tolerance (e.g., Baudier et al. 2015). Large size thus seems to provide fitness benefits in terms of increased survival. However, large individuals were also more strongly affected by desiccation stress. The latter conflicts with the findings of several studies in other systems suggesting that large individuals are more resistant to desiccation stress (Bujan et al. 2016; Chown and Nicolson 2004; Hadley 1994). One possibility is that behavioral responses and microhabitat choice are the main mechanisms that regulate desiccation stress in sepsids (as has been shown in other systems, e.g., Hood and Tschinkel 1990). Because our experimental setup limited behavioral responses, it is unclear whether the physiological responses that are detected under laboratory conditions are relevant in the field. Future research will be necessary to investigate the interactions between desiccation, plastic life history responses, and behavior under more natural conditions.

## 5 Conclusions

Agricultural landscapes are increasingly impacted by a variety of abiotic stressors. Although antiparasitic residues are well known to harm dung insects and disrupt their ecosystem functions, the role of ecological conditions in modulating these effects remains poorly understood. Here, we investigated the combined effects of ivermectin exposure, heat stress, desiccation stress, and the endogenous factors of sex and body size. We find that the combined effects of ivermectin exposure, desiccation, and heat stress have very strong but mostly additive (as opposed to synergistic) effects on survival. However, we also found that exposure to heat stress moderately reduced the lethality of ivermectin exposure, suggesting some cross‐resistance or hormesis. In addition, we find that the impacts of ivermectin on survival are especially strong in females compared to males. Taken together, our data suggest that previous research has overlooked important interactions between endogenous and external environmental factors that are likely to modulate the ecological impact of ivermectin (but see: González‐Tokman et al. 2022). Future research on more systems and more stressors will be necessary to fully understand how chemical residues impact ecosystem function in a dynamic world.

## Author Contributions

**Jill Walker:** conceptualization (equal), data curation (equal), formal analysis (equal), investigation (equal), methodology (equal), visualization (equal), writing – original draft (equal), writing – review and editing (equal). **Benjamin J. Mathews:** conceptualization (equal), formal analysis (equal), investigation (equal), methodology (equal), visualization (equal), writing – original draft (equal), writing – review and editing (equal). **Patrick T. Rohner:** conceptualization (equal), formal analysis (equal), funding acquisition (equal), investigation (equal), methodology (equal), project administration (equal), supervision (equal), visualization (equal), writing – original draft (equal), writing – review and editing (equal).

## Conflicts of Interest

The authors declare no conflicts of interest.

## Supporting information


**Appendix S1:** ece371929‐sup‐0001‐AppendixS1.docx.

## Data Availability

The data that support the findings of this study are deposited in Dryad at https://doi.org/10.5061/dryad.x0k6djhvc.

## References

[ece371929-bib-0001] Alvinerie, M. , J. F. Sutra , P. Galtier , et al. 1999. “Persistence of Ivermectin in Plasma and Faeces Following Administration of a Sustained‐Release Bolus to Cattle.” Research in Veterinary Science 66: 57–61. 10088713 10.1053/rvsc.1998.0240

[ece371929-bib-0002] Andreazza, F. , K. Haddi , S. D. Nörnberg , R. N. C. Guedes , D. E. Nava , and E. E. Oliveira . 2020. “Sex‐Dependent Locomotion and Physiological Responses Shape the Insecticidal Susceptibility of Parasitoid Wasps.” Environmental Pollution 264: 114605. 32380390 10.1016/j.envpol.2020.114605

[ece371929-bib-0003] Baudier, K. M. , A. E. Mudd , S. C. Erickson , and S. O'Donnell . 2015. “Microhabitat and Body Size Effects on Heat Tolerance: Implications for Responses to Climate Change (Army Ants: Formicidae, Ecitoninae).” Journal of Animal Ecology 84: 1322–1330. 26072696 10.1111/1365-2656.12388

[ece371929-bib-0004] Baur, J. , A. Giesen , P. T. Rohner , W. U. Blanckenhorn , and M. A. Schäfer . 2019. “Exaggerated Male Forelegs Are Not More Differentiated Than Wing Morphology in Two Widespread Sister Species of Black Scavenger Flies.” Journal of Zoological Systematics and Evolutionary Research 58: 159–173.

[ece371929-bib-0005] Blanckenhorn, W. U. , N. Puniamoorthy , M. A. Schäfer , A. Scheffczyk , and J. Römbke . 2013. “Standardized Laboratory Tests With 21 Species of Temperate and Tropical Sepsid Flies Confirm Their Suitability as Bioassays of Pharmaceutical Residues (Ivermectin) in Cattle Dung.” Ecotoxicology and Environmental Safety 89: 21–28. 23260241 10.1016/j.ecoenv.2012.10.020

[ece371929-bib-0006] Bueno, E. M. , C. L. McIlhenny , and Y. H. Chen . 2023. “Cross‐Protection Interactions in Insect Pests: Implications for Pest Management in a Changing Climate.” Pest Management Science 79: 9–20. 36127854 10.1002/ps.7191 PMC10092685

[ece371929-bib-0007] Bujan, J. , S. P. Yanoviak , and M. Kaspari . 2016. “Desiccation Resistance in Tropical Insects: Causes and Mechanisms Underlying Variability in a Panama Ant Community.” Ecology and Evolution 6: 6282–6291. 27648242 10.1002/ece3.2355 PMC5016648

[ece371929-bib-0008] Chown, S. , and S. W. Nicolson . 2004. Insect Physiological Ecology: Mechanisms and Patterns. OUP Oxford.

[ece371929-bib-0009] Conforti, S. , J. Dietrich , T. Kuhn , et al. 2018. “Comparative Effects of the Parasiticide Ivermectin on Survival and Reproduction of Adult Sepsid Flies.” Ecotoxicology and Environmental Safety 163: 215–222. 30055386 10.1016/j.ecoenv.2018.07.029

[ece371929-bib-0010] Correa, C. M. A. , K. R. Ferreira , A. R. Abot , J. Louzada , and F. Z. Vaz‐de‐Mello . 2022. “Ivermectin Impacts on Dung Beetle Diversity and Their Ecological Functions in Two Distinct Brazilian Ecosystems.” Ecological Entomology 47: 736–748.

[ece371929-bib-0011] Crump, A. 2017. “Ivermectin: Enigmatic Multifaceted ‘Wonder’ Drug Continues to Surprise and Exceed Expectations.” Journal of Antibiotics 70: 495–505. 28196978 10.1038/ja.2017.11

[ece371929-bib-0012] El‐Saber Batiha, G. , A. Alqahtani , O. B. Ilesanmi , et al. 2020. “Avermectin Derivatives, Pharmacokinetics, Therapeutic and Toxic Dosages, Mechanism of Action, and Their Biological Effects.” Pharmaceuticals (Basel) 13: 196. 32824399 10.3390/ph13080196 PMC7464486

[ece371929-bib-0013] Faly, L. , V. Brygadyrenko , A. Orzekauskaite , and A. Paulauskas . 2023. “Sensitivity of Non‐Target Groups of Invertebrates to Cypermethrin.” Biosystems Diversity 31: 393–400.

[ece371929-bib-0014] Fernandez, C. , M. S. Andrés , M. A. Porcel , C. Rodriguez , A. Alonso , and J. V. Tarazona . 2009. “Pharmacokinetic Profile of Ivermectin in Cattle Dung Excretion, and Its Associated Environmental Hazard.” Soil and Sediment Contamination 18: 564–575.

[ece371929-bib-0015] Gandara, L. , R. Jacoby , F. Laurent , et al. 2024. “Pervasive Sublethal Effects of Agrochemicals on Insects at Environmentally Relevant Concentrations.” Science 386: 446–453. 39446951 10.1126/science.ado0251

[ece371929-bib-0016] Ge, L.‐Q. , L.‐J. Huang , G.‐Q. Yang , et al. 2013. “Molecular Basis for Insecticide‐Enhanced Thermotolerance in the Brown Planthopper Ilaparvata Lugens Stål (Hemiptera:Delphacidae).” Molecular Ecology 22: 5624–5634. 24303791 10.1111/mec.12502

[ece371929-bib-0017] González‐Tokman, D. , S. S. Bauerfeind , M. A. Schäfer , R. J. Walters , D. Berger , and W. U. Blanckenhorn . 2022. “Heritable Responses to Combined Effects of Heat Stress and Ivermectin in the Yellow Dung Fly.” Chemosphere 286: 131030. 34144808 10.1016/j.chemosphere.2021.131030

[ece371929-bib-0018] Gould, S. J. , and E. S. Vrba . 1982. “Exaptation—A Missing Term in the Science of Form.” Paleobiology 8: 4–15.

[ece371929-bib-0019] Guedes, R. N. C. , R. R. Rix , and G. C. Cutler . 2022. “Pesticide‐Induced Hormesis in Arthropods: Towards Biological Systems.” Current Opinion in Toxicology 29: 43–50.

[ece371929-bib-0020] Hadley, N. F. 1994. The Water Relations of Terrestrial Arthropods. Academic Press.

[ece371929-bib-0021] Halsch, C. A. , D. J. Zullo , and M. L. Forister . 2023. “Additive and Interactive Effects of Anthropogenic Stressors on an Insect Herbivore.” Proceedings of the Royal Society B: Biological Sciences 290: 20222431. 10.1098/rspb.2022.2431 PMC10072940 37015275

[ece371929-bib-0022] Herd, R. P. , R. A. Sams , and S. M. Ashcraft . 1996. “Persistence of Ivermectin in Plasma and Faeces Following Treatment of Cows With Ivermectin Sustained‐Release, Pour‐On or Injectable Formulations.” International Journal for Parasitology 26: 1087–1093. 8982789

[ece371929-bib-0023] Hood, W. G. , and W. R. Tschinkel . 1990. “Desiccation Resistance in Arboreal and Terrestrial Ants.” Physiological Entomology 15: 23–35.

[ece371929-bib-0024] Jochmann, R. , and W. U. Blanckenhorn . 2016. “Non‐Target Effects of Ivermectin on Trophic Groups of the Cow Dung Insect Community Replicated Across an Agricultural Landscape.” Basic and Applied Ecology 17: 291–299.

[ece371929-bib-0025] Kavanaugh, B. , and P. Manning . 2020. “Ivermectin Residues in Cattle Dung Impair Insect‐Mediated Dung Removal but Not Organic Matter Decomposition.” Ecological Entomology 45: 671–678.

[ece371929-bib-0026] Liebig, M. , Á. A. Fernandez , E. Blübaum‐Gronau , et al. 2010. “Environmental Risk Assessment of Ivermectin: A Case Study.” Integrated Environmental Assessment and Management 6: 567–587. 20821718 10.1002/ieam.96

[ece371929-bib-0027] Losey, J. E. , and M. Vaughan . 2006. “The Economic Value of Ecological Services Provided by Insects.” Bioscience 56: 311–323.

[ece371929-bib-0028] Lumaret, J.‐P. , M. Alvinerie , H. Hempel , H.‐J. Schallnaß , D. Claret , and J. Römbke . 2007. “New Screening Test to Predict the Potential Impact of Ivermectin‐Contamined Cattle Dung on Dung Beetles.” Veterinary Research 38: 15–24. 17074292 10.1051/vetres:2006041

[ece371929-bib-0029] Madsen, M. , B. O. Nielsen , P. Holter , et al. 1990. “Treating Cattle With Ivermectin: Effects on the Fauna and Decompsition of Dung Pats.” Journal of Applied Ecology 27: 1–15.

[ece371929-bib-0030] McKellar, Q. A. 1997. “Ecotoxicology and Residues of Anthelmintic Compounds.” Veterinary Parasitology 72: 413–435. 9460209 10.1016/s0304-4017(97)00108-8

[ece371929-bib-0031] Nagloo, N. , E. Rigosi , L. Herbertsson , and D. C. O'Carroll . 2024. “Comparability of Comparative Toxicity: Insect Sensitivity to Imidacloprid Reveals Huge Variations Across Species but Also Within Species.” Proceedings. Biological Sciences 291: 20232811. 38864325 10.1098/rspb.2023.2811 PMC11285856

[ece371929-bib-0032] Neter, J. , M. H. Kutner , and W. Wasserman . 1985. Applied Linear Statistical Models. Irwin.

[ece371929-bib-0033] Pérez‐Cogollo, L. C. , R. I. Rodríguez‐Vivas , H. Delfín‐González , E. Reyes‐Novelo , and M. M. Ojeda‐Chi . 2015. “Lethal and Sublethal Effects of Ivermectin on *Onthophagus landolti* (Coleoptera: Scarabaeidae).” Environmental Entomology 44: 1634–1640, 1637. 26352254 10.1093/ee/nvv139

[ece371929-bib-0034] Pont, A. C. , and R. Meier . 2002. “The Sepsidae (Diptera) of Europe.” In Fauna Entomologica Scandinavica, vol. 37, 1–221. Brill.

[ece371929-bib-0035] Puniamoorthy, N. , M. A. Schäfer , J. Römbke , R. Meier , and W. U. Blanckenhorn . 2014. “Ivermectin Sensitivity Is an Ancient Trait Affecting All Ecdysozoa but Shows Phylogenetic Clustering Among Sepsid Flies.” Evolutionary Applications 7: 548–554. 24944568 10.1111/eva.12152 PMC4055176

[ece371929-bib-0036] Rathman, R. J. , M. W. Johnson , J. A. Rosenheim , B. E. Tabashnik , and M. Purcell . 1992. “Sexual Differences in Insecticide Susceptibility and Synergism With Piperonyl Butoxide in the Leafminer Parasitoid Diglyphus Begini (Hymenoptera: Eulophidae).” Journal of Economic Entomology 85: 15–20.

[ece371929-bib-0037] Rodrigues, Y. K. , and P. Beldade . 2020. “Thermal Plasticity in Insects' Response to Climate Change and to Multifactorial Environments.” Frontiers in Ecology and Evolution 8: 271.

[ece371929-bib-0038] Rodríguez‐Vivas, R. I. , G. S. Basto‐Estrella , E. Reyes‐Novelo , et al. 2020. “Evaluation of the Attraction, Lethal and Sublethal Effects of the Faeces of Ivermectin‐Treated Cattle on the Dung Beetle *Digitonthophagus gazella* (Coleoptera: Scarabaeidae).” Austral Entomology 59: 368–374.

[ece371929-bib-0039] Rohner, P. T. , Y. C. Ang , Z. Lei , N. Puniamoorthy , W. U. Blanckenhorn , and R. Meier . 2014. “Genetic Data Confirm the Species Status of Sepsis Nigripes Meigen (Diptera: Sepsidae) and Adds One Species to the Alpine Fauna While Questioning the Synonymy of Sepsis Helvetica Munari.” Invertebrate Systematics 28: 555–563.

[ece371929-bib-0040] Rohner, P. T. , and W. U. Blanckenhorn . 2018. “A Comparative Study of the Role of Sex‐Specific Condition Dependence in the Evolution of Sexually Dimorphic Traits.” American Naturalist 192: E202–E215. 10.1086/700096 30444660

[ece371929-bib-0041] Rohner, P. T. , W. U. Blanckenhorn , and N. Puniamoorthy . 2016. “Sexual Selection on Male Size Drives the Evolution of Male‐Biased Sexual Size Dimorphism via the Prolongation of Male Development.” Evolution 70: 1189–1199. 27168489 10.1111/evo.12944

[ece371929-bib-0042] Rohner, P. T. , J.‐P. Haenni , A. Giesen , et al. 2019. “Temporal Niche Partitioning of Swiss Black Scavenger Flies in Relation to Season and Substrate Age (Diptera, Sepsidae).” Alpine Entomology 3: 1–10.

[ece371929-bib-0043] Rohner, P. T. , and A. P. Moczek . 2023. “Allometric Plasticity and the Evolution of Environment‐By‐Environment (ExE) Interactions During a Rapid Range Expansion of a Dung Beetle.” Evolution 77: 682–689. 36626800 10.1093/evolut/qpac071

[ece371929-bib-0044] Rohner, P. T. , T. Teder , T. Esperk , S. Lüpold , and W. U. Blanckenhorn . 2018. “The Evolution of Male‐Biased Sexual Size Dimorphism Is Associated With Increased Body Size Plasticity in Males.” Functional Ecology 32: 581–591.

[ece371929-bib-0045] Schmidt, C. D. 1983. “Activity of an Avermectin Against Selected Insects in Aging Manure1.” Environmental Entomology 12: 455–457.

[ece371929-bib-0046] Selzer, P. M. , and C. Epe . 2021. “Antiparasitics in Animal Health: Quo Vadis?” Trends in Parasitology 37: 77–89. 33039282 10.1016/j.pt.2020.09.004

[ece371929-bib-0047] Sirois‐Delisle, C. , and J. T. Kerr . 2022. “Climate Change Aggravates Non‐Target Effects of Pesticides on Dragonflies at Macroecological Scales.” Ecological Applications 32: e2494. 34783410 10.1002/eap.2494

[ece371929-bib-0048] Skidmore, P. 1991. Insects of the British Cow‐Dung Community. Field Studies Council.

[ece371929-bib-0049] Stevenson, B. G. , and D. L. Dindal . 1987. “Functional Ecology of Coprophagous Insects: A Review.” Pedobiologia 30: 285–298.

[ece371929-bib-0050] Stillwell, R. C. , W. U. Blanckenhorn , T. Teder , G. Davidowitz , and C. W. Fox . 2010. “Sex Differences in Phenotypic Plasticity Affect Variation in Sexual Size Dimorphism in Insects: From Physiology to Evolution.” Annual Review of Entomology 55: 227–245. 10.1146/annurev-ento-112408-085500 PMC4760685 19728836

[ece371929-bib-0051] Sutton, G. , J. Bennett , and M. Bateman . 2014. “Effects of Ivermectin Residues on Dung Invertebrate Communities in a UK Farmland Habitat.” Insect Conservation and Diversity 7: 64–72.

[ece371929-bib-0052] Teder, T. , and T. Tammaru . 2005. “Sexual Size Dimorphism Within Species Increases With Body Size in Insects.” Oikos 108: 321–334.

[ece371929-bib-0053] Therneau, T. M. 2022. “coxme: Mixed Effects Cox Models. R Package Version 2.2–18.1.” https://CRAN.R‐project.org/package=coxme.

[ece371929-bib-0054] van Koppenhagen, N. , N. Gourgoulianni , P. T. Rohner , J. Roy , A. Wegmann , and W. U. Blanckenhorn . 2020. “Sublethal Effects of the Parasiticide Ivermectin on Male and Female Reproductive and Behavioural Traits in the Yellow Dung Fly.” Chemosphere 242: 125240. 31896183 10.1016/j.chemosphere.2019.125240

[ece371929-bib-0055] Verdú, J. R. , J. M. Lobo , F. Sánchez‐Piñero , et al. 2018. “Ivermectin Residues Disrupt Dung Beetle Diversity, Soil Properties and Ecosystem Functioning: An Interdisciplinary Field Study.” Science of the Total Environment 618: 219–228. 29128770 10.1016/j.scitotenv.2017.10.331

[ece371929-bib-0056] Villada‐Bedoya, S. , J. R. Chávez‐Ríos , B. Montoya , et al. 2021. “Heat Shock Proteins and Antioxidants as Mechanisms of Response to Ivermectin in the Dung Beetle *Euoniticellus intermedius* .” Chemosphere 269: 128707. 33168281 10.1016/j.chemosphere.2020.128707

[ece371929-bib-0057] Vogt, W. G. , T. L. Woodburn , B. A. Ellem , A. C. M. van Gerwen , L. B. Browne , and K. G. Wardhaugh . 1985. “The Relationship Between Fecundity and Oocyte Resorption in Field Populations of *Lucilia cuprina* .” Entomologia Experimentalis et Applicata 39: 91–99.

[ece371929-bib-0058] Yang, Y. , D. Tilman , Z. Jin , et al. 2024. “Climate Change Exacerbates the Environmental Impacts of Agriculture.” Science 385: eadn3747. 39236181 10.1126/science.adn3747

[ece371929-bib-0059] Zhang, Y. , Y. Wang , Z. Ma , D. Zhai , X. Gao , and X. Shi . 2019. “Cytochrome P450 Monooxygenases‐Mediated Sex‐Differential Spinosad Resistance in House Flies *Musca domestica* (Diptera: Muscidae).” Pesticide Biochemistry and Physiology 157: 178–185. 31153466 10.1016/j.pestbp.2019.03.024

[ece371929-bib-0060] Zhou, W. , M. Li , and V. Achal . 2025. “A Comprehensive Review on Environmental and Human Health Impacts of Chemical Pesticide Usage.” Emerging Contaminants 11: 100410.

