# Supplementary material for: Context‐Dependent Effects of Ivermectin Residues on Dung Insects: Interactions With Environmental Stressors, Size, and Sex in a Sepsid Fly (Sepsis neocynipsea)
Source: Ecol Evol. 2025 Aug 8;15(8):e71929. doi: 10.1002/ece3.71929 (PMC12334551; doi:10.1002/ece3.71929)
Supplement: Supplementary file 1 — Appendix S1: ece371929‐sup‐0001‐AppendixS1.docx. [file ECE3-15-e71929-s001.docx]

**Supplementary Material**


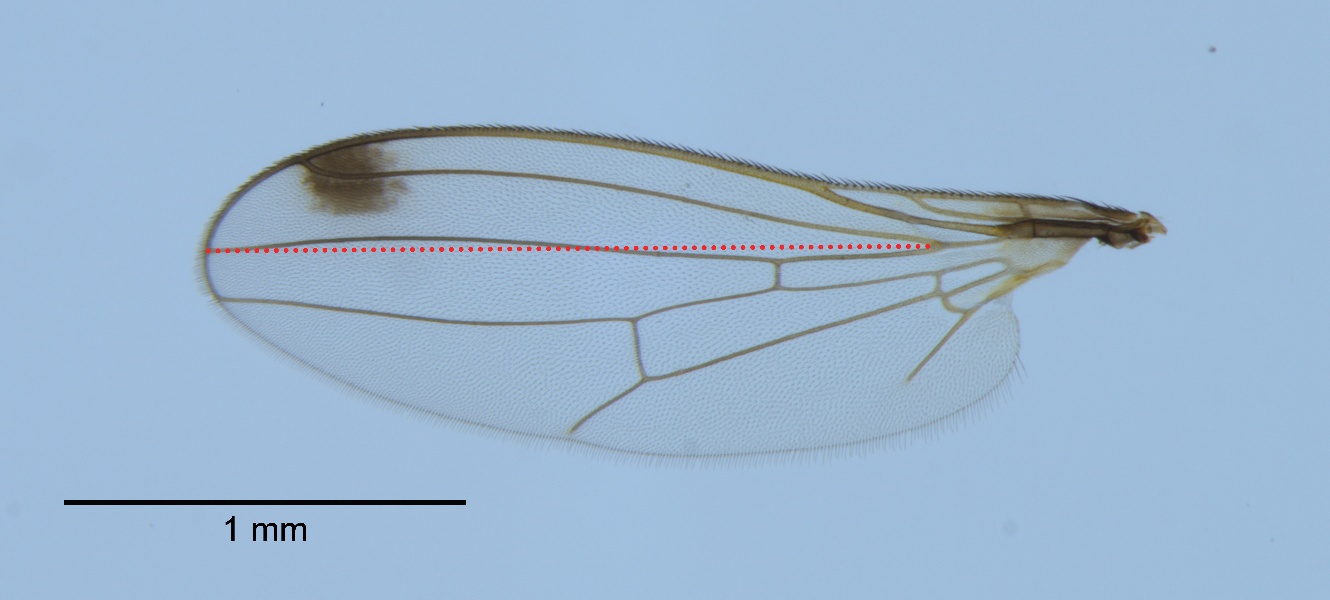


Figure S1: Wing morphology of Sepsis neocynipsea. The red dotted line indicates the measurement used to quantify wing length.

Table S1: Pearson’s correlation coefficients between wing length and six other measurements based on *Sepsis neocynipsea* individuals dissected and measured in (Rohner and Blanckenhorn 2018). The total sample size for the correlations is n = 69. Wing length correlates strongly with other linear morphological measurements, indicating that it is a good approximation of overall body size.

|  | Pearson's correlation coefficient (r) | 95% confidence limits |
| --- | --- | --- |
| wing width | 0.989 | [0.983, 0.993] |
| hind tibia length | 0.962 | [0.938, 0.976] |
| mid tibia length | 0.974 | [0.958, 0.984] |
| fore tibia length | 0.986 | [0.977, 0.991] |
| fore femur length | 0.961 | [0.938, 0.976] |
| thorax length | 0.981 | [0.969, 0.988] |
